# Inflammatory response in mixed viral-bacterial community-acquired pneumonia

**DOI:** 10.1186/1471-2466-14-123

**Published:** 2014-07-29

**Authors:** Salvador Bello, Elisa Mincholé, Sergio Fandos, Ana B Lasierra, María A Ruiz, Ana L Simon, Carolina Panadero, Carlos Lapresta, Rosario Menendez, Antoni Torres

**Affiliations:** 1 Servicio de Neumologia, Hospital Universitario Miguel Servet, Paseo Isabel La Católica, 1-3, 50009 Zaragoza, Spain; 2 Servicio de Bioquimica Clinica, Hospital Universitario Miguel Servet, Zaragoza, Spain; 3 Servicio de Microbiologia, Hospital Universitario Miguel Servet, Zaragoza, Spain; 4 Servicio de Medicina Preventiva, Hospital Universitario Miguel Servet, Zaragoza, Spain; 5 Servicio de Neumologia, ISS/Hospital Universitario y Politécnico La Fe CIBERES, Valencia, Spain; 6 Servicio de Neumologia, Hospital Clinic i Provincial de Barcelona – Institut d’Investigacions Biomediques August Pi i Sunyer (IDIBAPS), Universidad de Barcelona (UB) - Ciber de Enfermedades Respiratorias (Ciberes), Barcelona, Spain

**Keywords:** Community-acquired pneumonia, Viral pneumonia, Biomarkers

## Abstract

**Background:**

The role of mixed pneumonia (virus + bacteria) in community-acquired pneumonia (CAP) has been described in recent years. However, it is not known whether the systemic inflammatory profile is different compared to monomicrobial CAP. We wanted to investigate this profile of mixed viral-bacterial infection and to compare it to monomicrobial bacterial or viral CAP.

**Methods:**

We measured baseline serum procalcitonin (PCT), C reactive protein (CRP), and white blood cell (WBC) count in 171 patients with CAP with definite etiology admitted to a tertiary hospital: 59 (34.5%) bacterial, 66 (39.%) viral and 46 (27%) mixed (viral-bacterial).

**Results:**

Serum PCT levels were higher in mixed and bacterial CAP compared to viral CAP. CRP levels were higher in mixed CAP compared to the other groups. CRP was independently associated with mixed CAP. CRP levels below 26 mg/dL were indicative of an etiology other than mixed in 83% of cases, but the positive predictive value was 45%. PCT levels over 2.10 ng/mL had a positive predictive value for bacterial-involved CAP versus viral CAP of 78%, but the negative predictive value was 48%.

**Conclusions:**

Mixed CAP has a different inflammatory pattern compared to bacterial or viral CAP. High CRP levels may be useful for clinicians to suspect mixed CAP.

## Background

Viruses have only become known as a microorganism involved in CAP in the adult immunocompetent population in recent years. Available data show that a virus is the only microorganism isolated in between 10% and 30% of immunocompetent adults hospitalized for CAP, and accounts for as much as 40% of cases with established etiology. A bacterium as a co-pathogen of a virus can be found in association with CAP (mixed viral-bacterial pneumonia) [1-4] and this accounts for as much as 39% of cases in which an etiological agent is identified [1-3]. In these studies, *Streptococcus pneumoniae* continues to be the most frequent producer of adult CAP, though frequently in association with a co-pathogen, especially viruses (20%-40%) [2,3,5,6]. It seems logical that, due to the lower sensitivity of techniques usually employed to diagnose bacterial infection, some pneumonias considered to be viral will actually be mixed CAP [3]. This issue, together with the possible isolation of viruses from asymptomatic subjects, suggesting clearance of a past or current subclinical upper airway infection not related to a concomitant CAP, has led to uncertainty as to whether some viruses are the real cause of CAP or whether they must act as a co-pathogen with a bacteria in adults [2,3,6-8]; the generally accepted opinion is that viruses other than influenza rarely cause pneumonia in healthy adults [9].

Besides individual host responses, there is increasing evidence that several different causal microorganisms may trigger different inflammatory responses, and levels of several markers such as white blood cells (WBC), C-reactive protein (CRP) and procalcitonin (PCT) are associated with different etiological patterns [10,11]. Clinical signs and symptoms of bacterial and viral pneumonia are highly variable and overlap, and there is no clinical or radiologic algorithm that can discriminate between the two causes of CAP [12]. Some attempts at differentiation based on biomarkers have been made, and it is now well established that CRP and, especially, PCT show higher levels in bacterial than in viral (or atypical) pneumonia [12-14]. Lack of viral PCT response to viral infection is thought to be related to post-infection release of interferon, which inhibits PCT synthesis [15].

There are very few studies of mixed viral-bacterial CAP that involve biomarkers. The only available data concern H1N1 influenza, from retrospective studies with a limited number of patients, mostly in those admitted to the ICU, showing increased PCT [16-19] and CRP [18,19] levels in bacterial coinfection; this suggests that the biomarkers may be used as a tool for discriminating mixed CAP from H1N1 viral CAP. The CAPNETZ study included a large number of patients (1337) with CAP, of which 58 (4.3%) showed mixed etiologies; however, this group was heterogeneous, as it included two or more typical or atypical bacteria and combinations of typical with atypical and any bacteria with a virus [10].

We wanted to determine WBC, CRP and PCT levels in mixed viral-bacterial CAP, and wondered whether biomarkers in mixed disease showed a different pattern than in either bacterial or viral CAP alone.

## Methods

### Ethics statement

This study received written approval from the Instituto Aragonés de Ciencias de la Salud (IACS) review board.

### Study design

From February 2009 to December 2010 (22 months), consecutive adult (>18 years) patients admitted to a university hospital with a diagnosis of CAP were prospectively recruited within 24 hours of their arrival. CAP was defined as an acute disease with a new radiologic infiltrate not due to another known cause, in association with symptoms of lower respiratory-tract infection. Exclusion criteria were severe immunodepression (HIV infection, severe hematological disease); immunosuppressive therapy (prednisone or equivalent daily dose of >20 mg for >2 weeks), or any immunosuppressive regimen (azathioprine, cyclosporine, cyclophosphamide and/or other immunosuppressant drugs); leucopenia < 1000/mm^3^ or neutropenia <500/mm^3^ and/or chemotherapy in the previous year; pulmonary abscess (x-ray cavitation), aspiration pneumonia and obstructive pneumonia; possible nosocomial origin (less than 30 days after hospital discharge); and known active neoplasia. The study was approved by the ethics committee and all patients signed an informed consent form. All patients were followed up during their hospital stay and those with a definitive diagnosis other than CAP were excluded.

Throughout the study period, a sex-matched and age-matched control group was collected from subjects admitted to the orthopedic-surgery, digestive medicine, and neurology departments. None of these patients had had any suspected infectious or respiratory disease in the previous two months, immunosuppression, known neoplasia, or recent trauma, and none had recently undergone surgical procedures. The objectives of this control group were to determine the false positive rate of our viral PCR techniques in the same period than our CAP group, as well as to compare biomarker levels in both populations.

The following variables were recorded: age, sex, anti-influenza vaccination <1 year, antipneumococcal vaccination <5 years, tobacco (non-smoker, ex-smoker, pack-years) alcohol (non-drinker, ex-drinker, current drinker [<80 g/day, >80 g/day]), comorbidities, previous pneumonia, recent (1 month) antibiotic treatment. On admission: days of duration of disease, symptoms prior to infection, myalgia, CAP signs and symptoms, vital constants on admission to the emergency department (ED) (respiratory and heart rates, arterial pressure, SaO_2_), number of hours in the ED, arterial blood gas determinations (when performed), number of lobes involved and type of x-ray condensation (alveolar, interstitial, mixed), and pneumothorax/atelectasis/bleeding. Pneumonia Severity Index (PSI) and the severity score from British Thoracic Society (CURB65) were calculated for all patients. All patients were admitted to hospital for at least 24 hours.

#### Determination of leukocyte count, CRP and PCT

Venous blood samples were collected from CAP patients and controls on admission to the ED, within 6 hours of arrival. A sample was submitted to the hematology lab for a WBC count, and another sample with EDTA was submitted to the biochemistry lab for CRP and PCT assessment. These latter samples were centrifuged and stored at −80°C until biomarker tests were performed. Serum CRP was measured by means of immunoturbidimetry using the highly sensitive near-infrared particle immunoassay (NIPIA) method (IMMAGE 800, BeckmanCoulter, San Diego, USA). The assay has an analytical detection limit of 0.06 mg/L and a functional assay sensitivity of 0.11 mg/L. Procalcitonin concentrations were determined using sandwich immunoassays and time-resolved amplified cryptate emission (TRACE) measurement (PCT sensitive KRYPTOR, BRAHMS, Hennigsdorf, Germany), as described in detail previously [20]. The analytical detection limit and the functional assay sensitivity for the assays were 0.02 ng/mL and 0.06 ng/mL, respectively, for procalcitonin. Measurements of PCT were performed in our laboratory in a blinded fashion without knowing the clinical parameters and microbiological results.

#### Microbiological tests

Blood was drawn from CAP patients in the ED shortly after CAP diagnosis for bacterial culture. Sputum was obtained when possible and before antibiotics were administered, and immediately sent for Gram staining and culture; only samples containing a preponderance of leukocytes and a few squamous epithelial cells were considered acceptable. Another blood sample was obtained for *Mycoplasma pneumoniae* (Complement Fixation test, Virion Serion Institut, Würzburg, Germany) and *Chlamydophila pneumoniae* (ELISA test, Savyon Diagnostics Ltd, St. Ashdod, Israel) first serum test. Second serological tests were performed on blood obtained during the 30-day follow-up visit, when possible. Urine was taken in the first 24 hours and tested for *Streptococcus pneumoniae* and *Legionella pneumophila* antigens (BINAX now, Binax, Portland, ME, USA).

Nasopharyngeal aspirate was obtained and processed for viral antigens using the direct fluorescence antibody (DFA) assay, and two different polymerase chain reaction (PCR) viral tests. DFA was performed for influenza A and B, parainfluenza 1, 2 and 3, adenovirus (ADV), human metapneumovirus (hMPV) and respiratory syncytial virus (RSV) (D3 Double Duet DFA Respiratory virus screening and ID Kit, Diagnostic Hybrids, Athens, USA). The first PCR was a multiplex RT-nested PCR assay for 14 respiratory viruses (influenza A, B and C, respiratory syncytial virus A and B, adenovirus, coronavirus 229E and OC43, enterovirus, parainfluenza 1, 2, 3 and 4, and rhinovirus) [21]; the second test was a RT-PCR commercial kit, the ResPlex II Plus Panel (Qiagen LiquiChip System, Hamburg, Germany) [22] for detection of 18 viruses: influenza A and B, RSV A and B, parainfluenza 1, 2, 3 and 4, hMPV A and B, enterovirus (coxsackie/echovirus), rhinovirus, adenovirus B and E, coronavirus NL63, HKU1, 229E and OC43, and bocavirus. Nucleic acids were extracted from nasopharyngeal aspirates immediately after their reception.

After excluding atypical-involved CAP (*M pneumoniae* and *C pneumoniae*), patients with CAP were divided into 3 groups: bacterial, viral and mixed. Patients with no microbiological findings were considered as CAP of unknown cause.

**Figure 1:**
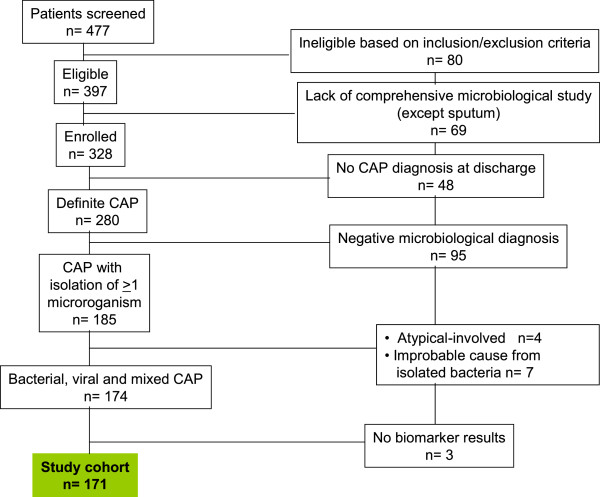
**Flow diagram for patient enrollment or exclusion in the study.** After excluding patients who did not meet the inclusion criteria, those with no comprehensive microbiological study, those with a final diagnosis other than CAP, and those with negative microbiological diagnosis, we obtained 185 patients with CAP and isolation of at least 1 microorganism. We subsequently excluded 4 patients with atypical bacteria-involved CAP, all of which were Mycoplasma pneumoniae (1 M pneumoniae + E coli, 1 M pneumoniae + influenza A, 1 M pneumoniae + influenza A + influenza B + syncytial respiratory virus [RSV], and 1 M pneumoniae isolated), and a further 7 due to a bacterial yield of improbable CAP cause because of their low pathogenicity (2 from bacterial group [1 Enterococcus faecalis and 1 Staphylococcus hominis) and 5 from mixed (2 S. hominis + Adenovirus, 1 S. hominis + Virus influenza A + RSV, 1 Staphylococcus coagulase-negative + metapneumovirus, and 1 Morganella morgagnii + coronavirus]). We then had 174 patients with viral, bacterial and mixed pneumonia. A biomarker search could not be performed in three patients, and we finally included 171 patients in our study with both etiology and biomarkers.

*S pneumonia*e, other potentially pathogen gram-positive and gram-negative bacteria classically considered producers of CAP (*Haemophilus influenzae, Moraxella catarrhalis, Staphylococcus aureus*, etc.) and *L pneumophila,* were included in the group of “bacterial” CAP. One or more typical bacteria (including *Legionella*) in association with one or more virus were considered as mixed CAP.Viral CAP was defined as a CAP with detection of a virus and no isolation of bacteria or atypicals. Overall viral and bacterial was the result of adding viral and bacterial groups of CAP. Bacterial-involved was the addition of mixed (bacterial and viral) and bacterial CAP. Both positive microbiological findings and biomarker test were required to include patients in the study group (Figure 1).

### Analysis

Descriptive analysis data are presented as n (%) for categorical variables, and as median (interquartile range [IQR]) for continuous variables (after non-normal distribution was shown), as appropriate. Baseline characteristics of CAP patients were compared among the three groups together using ANOVA analysis or Kruskal-Wallis H test depending on the type of variable. Comparisons, except for baseline characteristics, were established between two different groups. In order to compare the values of every biomarker between the different etiologic groups, two-group comparisons were performed using the Mann–Whitney *U*-test. For every biomarker the two-group comparisons performed were: bacterial vs mixed; bacterial vs viral; mixed vs viral; mixed vs overall bacterial and viral; and mixed vs unknown. P-values <0.05 were considered to indicate statistical significance.

A large number of univariate and multivariate logistic regression analyses were performed to investigate the factors associated with mixed viral-bacterial CAP, including age, comorbidities (renal failure, chronic liver disease, diabetes, COPD), clinical (fever, dyspnea, pleuritic pain, purulent sputum) and radiological data (type of infiltrate, number of lobes involved), severity scores (PSI and CURB65), and biomarkers (PCT, CRP and WBC). The best model was selected by a stepwise procedure.

We constructed receiver operating characteristic (ROC) curves and determined the area under the curve (AUC). The AUC and its 95% confidence intervals were estimated for each biomarker to predict different CAP etiologies (bacterial, viral, mixed, and bacterial-involved) and compared using a nonparametric method. Sensitivity, specificity, positive and negative predictive values, and positive and negative likelihood ratios (LHR + and LHR-, respectively) were calculated for each cut-off value of the biomarkers.

Statistical analysis was performed using the SPSS statistical software package, version 15.0. The level of significance for all statistical tests was a 2-sided, p value of 0.05.

## Results

### Patients and controls

We selected 280 patients from those admitted to the emergency department due to CAP, after excluding those who did not meet inclusion criteria, those without a comprehensive microbiological study and those with a final diagnosis other than CAP on discharge. A total of 185 cases were finally selected. The Figure 1 shows a flowchart explaining the process of enrollment of patients. Our final three groups were as follows: 59 (34.6%) bacterial, 66 (38.5%) viral and 46 (26.9%) mixed viral-bacterial CAP, and their main clinical characteristics are summarized in Table 1. Ninety five patients had a negative microbiological study, and 3 of them had no biomarker results (92 of unknown cause).

**Table 1:** Baseline characteristics of the 171 cases of community-acquired pneumonia included in the study

**Characteristic**		**Bacterial n = 59**	**Viral n = 66**	**Mixed n = 46**	**p**
**Age, years**		72 (14)	76 (17)	72 (20)	NS
**>65 years**		42 (70%)	50 (75.76%)	32 (68.09%)	NS
**Male sex**		42 (70%)	40 (60.61%)	23 (48.94%)	NS
**Smoking status**	**Yes**	16 (27.12%)	11 (16.67%)	9 (19.15%)	NS
	**No**	17 (28.81%)	33 (50%)	22 (46.81%)	NS
	**Ex-smoker**	26 (44.07%)	22 (33.33%)	16 (34.04%)	NS
**Previous antibiotic**		12 (20%)	16 (24.24%)	6 (12.77%)	NS
**Concomitant illnesses**	**Neoplastic disease (not active)**	7 (11.67%)	10 (15.15%)	5 (10.64%)	NS
	**Heart disease**	25 (41.67%)	23 (34.85%)	14 (29.79%)	NS
	**Cerebrovascular disease**	11 (18.33%)	14 (21.21%)	7 (14.89%)	NS
	**COPD**	21 (35%)	19 (28.79%)	12 (25.53%)	NS
	**Liver disease**	2 (3.33%)	2 (3.03%)	1 (2.13%)	NS
	**Renal disease**	6 (10%)	9 (13.64%)	5 (10.64%)	NS
	**Diabetes mellitus**	11 (18.33%)	15 (22.73%)	6 (12.77%)	NS
	**Bronchiectasis**	5 (8.33%)	1 (1.52%)	2 (4.26%)	NS
	**No first pneumonia**	20 (33.33%)	13 (19.7%)	12 (25.53%)	NS
**Clinical findings**	**Duration of symptoms prior to enrolment (mean no. of days)**	3	3,75	3,25	NS
	**Altered mental status**	5 (8.33%)	10 (15.15%)	2 (4.26%)	NS
	**Pulse > 125/min**	9 (15%)	4 (6.06%)	2 (4.26%)	NS
	**Respiratory rate > 30/min**	16 (26.67%)	10 (15.15%)	5 (10.64%)	NS
	**Systolic BP < 90 mmHg or diastolic BP < 60 mmHg**	11 (18.33%)	6 (9.09%)	5 (10.64%)	NS
	**Temperature < 35°C or > 40°C**	3 (5%)	1 (1.52%)	2 (4.26%)	NS
**Radiographic findings**	**Unilobar involvement**	51 (86.44%)	58 (89.23%)	39 (82.98%)	NS
	**Multilobar/bilateral involvement**	8 (13.66%)	8 (10.76%)	7 (17.02%)	NS
	**Pleural effusion**	15 (25%)	11 (16.67%)	12 (25.53%)	NS
**PSI**	**Mean PSI score**	101.5	106	101	NS
	**Class 1-3**	22 (37.3%)	23 (34.8%)	19 (41.3%)	NS
	**Class 2-5**	37 (62.7%)	43 (65.2%)	27 (58.7%)	
**CURB65**	**0-1**	23 (39%)	24 (36.4%)	19 (41.3%)	NS
	**2-5**	36 (61%)	42 (63.6%)	27 (58.7%)	
**Symptoms**	**Fever**	45 (77.59%)	46 (71.88%)	35 (76.09%)	NS
	**Cough**	44 (73.33%)	53 (79.1%)	39 (82.98%)	NS
	**Expectoration**	40 (67.8%)	47 (73.44%)	35 (77.78%)	NS
	**Dyspnea**	39 (67.24%)	48 (73.85%)	31 (67.39%)	NS
	**Pleuritic pain**	27 (46.55%)	23 (37.1%)	28 (63.64%)	0.026
	**Digestive**	9 (15.52%)	8 (12.9%)	12 (26.09%)	NS
	**Headache**	5 (20%)	7 (22.58%)	8 (42.11%)	NS
	**Myalgia**	4 (16.67%)	8 (27.59%)	7 (36.84%)	NS

Patients were distributed, according to PSI and CURB65 scores, as severe (PSI 4–5 and/or CURB65 2–5) and non-severe (PSI 1–3 and/or CURB65 0–1). Severe bacterial CAP included 38/59 (PSI) and 36/59 (CURB65); viral CAP, 43/66 (PSI) and 40/66 (CURB65); and mixed, 28/46 (PSI) and 29/46 (CURB65) patients. No differences were found for either clinical score (p = 0.831 for PSI and 0.950 for CURB65) when proportions of severe and non-severe CAP were compared in the three groups.After excluding atypical and low-pathogenicity bacteria, we selected 174 patients with viral, bacterial and mixed CAP. A biomarker search could not be performed in 3 patients, and we finally included 171 (61%) patients in our study with both etiology and biomarkers (see Figure 1). Of these, one patient from the viral group and another from the mixed group lacked PCT determination.

### Microbial etiology

Etiological agents isolated in each group are shown in Table 2. With the exception of atypical serology, every patient underwent all diagnostic tests. *Streptococcus pneumoniae* was the most frequent agent associated with CAP [67/280, 23.9% (67/171, 39.2% of CAP of known etiology)] and was found in 34/59 (57.6%) patients in the bacterial group: 29 as a single microorganism and 5 in association with other bacteria. It was also identified in 33/46 (71.7%) of mixed bacterial/viral etiologies, in which the most prevalent associations were *S pneumoniae* with rhinovirus (12), influenza A (10), and adenovirus (8). In the viral CAP group, the most frequently isolated pathogens were Influenza A (21), adenovirus (17) and rhinovirus (15). Of 67 cases of pneumonia in which S *pneumoniae* was isolated, 33/67 (49.2%) were associated with viruses.

**Table 2:** Microbial etiologies of the 171 cases of CAP included in the study

**Bacterial**	**n:**	**Viral**	**n:**	**Mixed**	**n:**
*S pneumoniae*	29	Adenovirus	10	*S pneumoniae* + Adenovirus	5
*E coli*	3	Influenza virus A	9	*S pneumoniae* + Rhinovirus	6
*Legionella pneumophila*	4	Rhinovirus	9	*S pneumoniae* + Influenza A	4
SARM	3	Coronavirus	7	*S pneumoniae* + Metapneumovirus	2
*H influenzae*	2	RSV-A	3	*S pneumoniae* + Influenza B	1
*Corynebacterium striatum*	2	RSV-B	1	*S pneumoniae* + *Moraxella catarrhalis* + Influenza virus A	1
*Serratia marcescens*	1	Influenza virus B	1	*S pneumoniae* + *S aureus* + Adenovirus	1
*Moraxella catarrhalis*	1	Metapneumovirus	1	*S pneumoniae* + *H influenzae* + Adenovirus + Rhinovirus + Cox virus	1
*Alcaligenes xylosoxidans*	1	Influenza virus A + Influenza virus B	2	*S pneumoniae* + Rhinovirus + Influenza A	1
*Enterobacter cloacae*	1	Influenza virus A + Parainfluenza 1	1	*S pneumoniae* + Rhinovirus + Cox virus	2
*S pneumoniae* + *H influenzae*	2	Influenza virus A + RSV-A	1	*S pneumoniae* + Rhinovirus + H1N1	1
*S pneumoniae* + *S aureus*	2	Influenza virus A + H1N1	1	*S pneumoniae* + Adenovirus + Coronavirus	1
*S pneumoniae* + *P aeruginosa*	1	Influenza virus A + Coronavirus	1	*S pneumoniae* + Influenza virus A + Parainfluenza 4	1
*P aeruginosa* + *Legionella*	1	Influenza virus A + Rhinovirus	1	*S pneumoniae* + Influenza virus A + RSVA	1
P aeruginosa + *C striatum*	1	Influenza virus A + Adenovirus	1	*S pneumoniae* + Influenza virus A + Coronavirus	1
*P aeruginosa* + *S marcescens* + *H influenzae*	1	Influenza virus A + RSV + Metapneumovirus	2	*S pneumoniae* + Rhinovirus + Coronavirus + Enterovirus	1
*E coli* + *Corynebacterium striatum* + *A baumannii*	1	Influenza virus A + RSV + V. influenza B	1	*S pneumoniae* + Coronavirus + Influenza v. A + Influenza v. B + RSV	1
*E coli* + *Achromobacter xylosoxidans*	1	Adenovirus + RSV-A	1	*S pneumoniae* + RSVB	2
*S aureus* + *Enterococcus faecium*	1	Adenovirus + Parainfluenza 3	1	*H influenzae* + Parainfluenza 3	1
*S aureus* + *Pasteurella multocida*	1	Adenovirus + Rhinovirus	1	*H influenzae* + Adenovirus + H1N1	1
		Adenovirus + Coronavirus	1	*H influenzae* + Adenovirus + Rhinovirus	1
		Adenovirus + Influenza virus B + RSV	1	*H influenzae* + Rhinovirus + RSVA	1
		Adenovirus + Enterovirus + Coronavirus	1	*P aeruginosa* + H1N1	1
		Rhinovirus + Influenza virus B	1	*P aeruginosa* + Rhinovirus	1
		Rhinovirus + Cox virus	3	*P aeruginosa* + RSVA + Coronavirus	1
		Coronavirus + RSV-A	2	*P aeruginosa* + RSVB	1
		Parainfluenza 1 + H1N1	1	*E coli* + Influenza virus B + Rhinovirus	1
		Cox virus + Enterovirus	1	*E coli* + Influenza virus A + RSV	1
				*Moraxella catarrhalis* + Coronavirus	1
				*Corynebacterium striatum* + Metapneumovirus	1
				*Stenotrophomonas maltophilia* + Adenovirus	1
**TOTAL**	**59**	**TOTAL**	**66**	**TOTAL**	**46**

Viruses were involved in 40% [(112/280) and 65.5% (112/171) of cases of CAP with established cause (excluding atypical)], whereas 46/280 (16.4%) and 46/171 (26.9%) of those of known etiology had a mixed bacterial-viral etiology. This information is summarized in Table 2.

The control group was composed of 100 subjects matched for age and sex. Comorbidities in this group were similar to those of CAP patients. Biomarker levels were checked in all of them. Nasopharyngeal aspirates were obtained in 60 of the subjects for viral searches, including both PCR tests. Paired blood samples for serological studies were not obtained in these subjects. In the control group, 4 viruses (3 adenovirus and 1 parainfluenza 4) were isolated in 4 subjects (4/60, 6.7%). PCT, CRP and WBC were significantly higher in CAP patients than in the controls, as shown in Table 3.

**Table 3:** Median (interquartile range) levels of biomarkers in CAP and control groups

**Biomarkers**	**PCT**	**CRP**	**WBC**
**Cohort**	**(ng/mL)**	**(mg/dL)**	**(10**^**3**^**/μL)**
**Control**	0.06 (0.08)	1.60 (6.61)	7.90 (3.10)
	(n = 100)	(n = 100)	(n = 97)
**CAP**	0.90 (4.35)	18.50 (21.92)	12.60 (8.20)
	(n = 169)	(n = 171)	(n = 171)
**p-value**	<0.001	<0.001	<0.001

### Biomarkers and etiology

**Figure 2:**
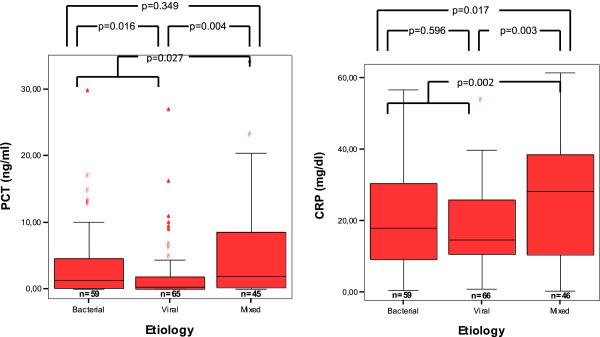
Median procalcitonin (PCT) and C reactive protein (CRP) values for bacterial, viral and mixed CAP.

Bacterial and mixed CAP showed no differences for PCT (p = 0.416). Viral CAP had significantly lower PCT values than both bacterial and mixed (p = 0.02 and 0.007, respectively). CRP was significantly higher in mixed CAP than in bacterial (p = 0.027) and viral (p = 0.005) CAP, and there were no differences between the latter two groups (p = 0.614). Both PCT and CRP showed significantly higher values in mixed group compared to overall viral and bacterial CAP, and to those with unknown cause. WBC counts in bacterial, mixed and viral CAP showed no differences among the three groups (p > 0.05) (Table 4). See Figure 2.

**Table 4:** Biomarker values in different etiologic groups of CAP

	**Bacterial**			**Mixed**			**Viral**			**Unknown**			**p**				
	**N**	**Median**	**Interquartile range**	**N**	**Median**	**Interquartile range**	**N**	**Median**	**Interquartile range**	**N**	**Median**	**Interquartile range**	**Bacterial vs Mixed**	**Bacterial vs Viral**	**Mixed vs Viral**	**Mixed vs Overall bacterial and viral**	**Mixed vs Unknown**
**PCT (ng/mL)**	59	1,37	4,635	45	1,978	8,324	65	0,38	1,756	92	0.18	0.899	0,416	0,02	0,007	0,044	0.002
**CRP (mg/dL)**	59	18	21,64	46	28,19	28,15	66	14,535	15,14	92	17.10	18.165	0,027	0,614	0,005	0,004	0.001
**WBC (cells/mm**^**3**^**)**	59	13,4	7,2	46	12,05	8,7	66	11,6	8,1	92	11.90	7.900	0,235	0,109	0,866	0,588	0.676

PCT, CRP and WBC showed similar results in influenza CAP and non-influenza viral CAP (p = 0.299, 0.345 and 0.091, respectively) (Table 5). When influenza A and B were removed from viral (n = 25) and mixed (n = 15) groups, PCT and CRP levels showed significantly higher levels in mixed CAP compared to viral CAP without influenza (p = 0.019 and 0.046, respectively). WBC showed no differences in either biomarker (Table 6). CRP levels were also higher in mixed involving influenza than in viral CAP due to influenza virus (Table 7).

**Table 5:** Biomarkers values in influenza and viral without influenza CAP

**Biomarkers**	**Viral without influenza**			**Influenza**			**p**
	**N**	**Median**	**Interquartile range**	**N**	**Median**	**Interquartile range**	
PCT (ng/mL)	40	0,388	1,656	25	0,380	4,285	0,299
CRP (mg/dL)	41	14,200	13,880	25	15,580	13,720	0,345
WBC (cells/mm^3^)	41	12,900	7,600	25	10,700	6,700	0,091

**Table 6:** Biomarker values in mixed and viral CAP, after removing influenza virus

	**Mixed without influenza**			**Viral without influenza**			**p**
	**N**	**Median**	**Interquartile range**	**N**	**Median**	**Interquartile range**	
PCT (ng/mL)	30	2,0545	9,407	40	0,388	1,656	0,019
CRP (mg/dL)	31	26,8	31,4	41	14,200	13,880	0,046
WBC (cells/mm^3^)	31	12,7	10,4	41	12,900	7,600	0,798

**Table 7:** Biomarker values in influenza and mixed with influenza CAP

	**Influenza**			**Mixed with influenza**			**p**
	**N**	**Median**	**Interquartile range**	**N**	**Median**	**Interquartile range**	
PCT (ng/mL)	25	0,376	4,674	15	1,880	4,001	0,315
CRP (mg/dL)	25	15,580	13,720	15	31,710	24,600	0,035
WBC (cells/mm^3^)	25	10,700	6,700	15	11,100	9,000	0,679

To differentiate mixed from overall bacterial and viral CAP, a cut-off of CRP of 25.95 mg/dL, near our median values, showed a PPV of 0.45 (95% CI, 0.32-0.58) and an NPV of 0.83 (0.76-0.9).

With regard to PCT, a cut-off of 2.10 ng/mL (near our median values) discriminated bacterial-involved (bacterial and mixed) from viral CAP with a positive predictive positive value (PPV) of 0.78 (95% confidence interval [CI], 0.68-0.88) and a negative predictive value (NPV) of 0.48 (95% CI, 0.38-0.57).

A large number of univariate and multivariate logistic regression analyses were performed to investigate the factors associated with mixed viral-bacterial CAP. The final multivariate logistic regression model selected by a stepwise procedure included only one independent variable, the CRP that was the only independent factor associated with mixed etiology. The other parameters showed no association with viral-bacterial CAP after adjusting for the rest of variables (Table 8).

**Table 8:** Uni and multivariate logistic regression analysis for mixed etiology

**Variable**	**Univariate**				**Multivariate**			
	p -value*	**OR**	**IC 95%**		p -value*	**OR**	**IC 95%**	
Age	0,447	0,992	0,971	1,013				
Sex	0,055	0,514	0,261	1,014				
PSI	0,568	0,819	0,412	1,628				
CURB65	0,789	1,100	0,547	2,211				
Diabetes	0,240	0,563	0,216	1,469				
Chronic hepatopathy	0,716	0,663	0,072	6,089				
Chronic renal failure	0,817	0,881	0,301	2,575				
Heart failure	0,311	0,689	0,335	1,418				
Cerebrovascular disease	0,456	0,707	0,283	1,765				
No. Of days of symptom	0,772	1,010	0,945	1,080				
Fever	0,842	1,084	0,492	2,390				
Purulent sputum	0,122	1,751	0,861	3,561				
Dyspnea	0,674	0,855	0,413	1,772				
Pleuritic pain	0,014	2,450	1,200	5,000	>0.05			
No. lobes involved	0,401	1,491	0,587	3,788				
COPD	0,429	0,737	0,346	1,569				
PCT (ng/ml)	0,019	1,069	1,011	1,130	>0.05			
CRP (mg/dl)	0,002	1,040	1,014	1,067	0,002	1,040	1,014	1,067
WBC	0,966	0,999	0,949	1,051				

**Figure 3:**
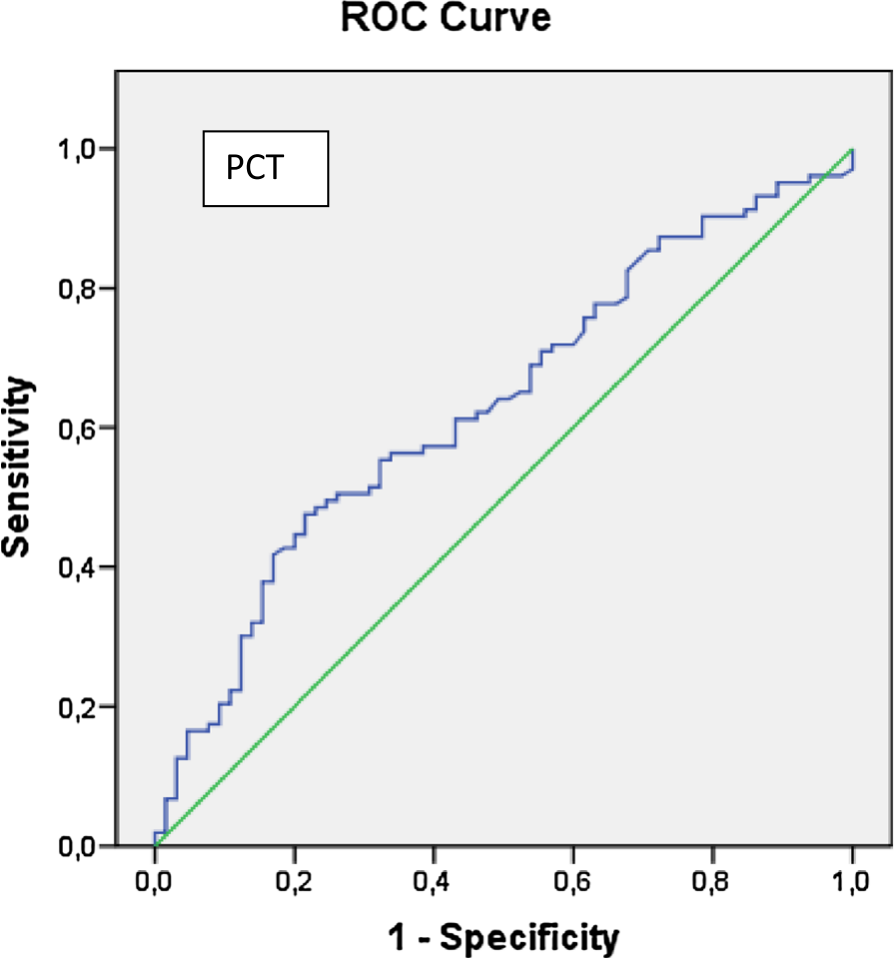
**Receiving operating characteristic curve of PCT for differentiating bacterial-involved (bacterial and mixed) from viral CAP.** AUC: 0.640, 95% CI: 0.557-0.723. p = 0.002.

**Figure 4:**
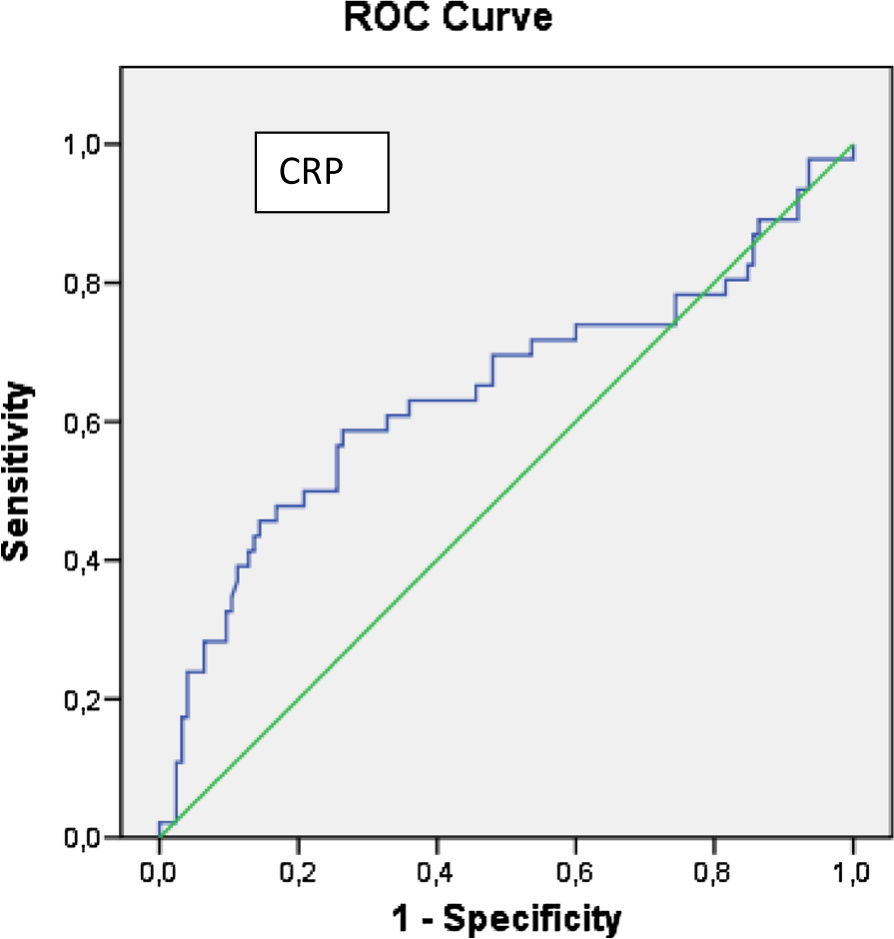
**Receiving operating characteristic curve of CRP for differentiating mixed from bacterial and viral CAP.** AUC: 0.642, 95% CI: 0.537-0.747. p = 0.004.

Receiver operating characteristic (ROC) analysis for PCT to discriminate bacterial-involved (bacterial and mixed) from viral CAP showed an area under curve (AUC) of 0.640 (p = 0.002). The AUC was 0.621 (p = 0.02) for discriminating between bacterial and viral CAP, and 0.651 (p = 0.007) for discriminating between mixed and viral CAP. The CRP AUC for discriminating between bacterial-involved and viral CAP was not significant (0.579, p = 0.081), but there were differences between mixed and bacterial (0.626, p = 0.027), mixed and viral (0.657, p = 0.005), and mixed and overall bacterial and viral (0.642, p = 0.004) (Table 9). See Figures 3 and 4.

**Table 9:** Results of receiver operating characteristic (ROC) analysis for PCT and CRP for discrimination among etiologies

**PCT**	**AUC**	**95% CI**	**Significance**
Bacterial-involved (bacterial and mixed) vs viral	0.640	0.557-0.723	p = 0.002
Bacterial vs viral	0.621	0.522-0.720	p = 0.02
Mixed vs viral	0.651	0.544-0.758	p = 0.007
**CRP**	**AUC**	**95% CI**	**Significance**
Bacterial-involved (bacterial and mixed) vs viral	0.579	0.494-0.665	NS
Mixed vs bacterial	0.626	0.514-0.738	p = 0.027
Mixed vs viral	0.657	0.546-0.768	p = 0.005
Mixed vs overall bacterial and viral	0.642	0.537-0.747	p = 0.004

## Discussion

The main conclusions of our study are that biomarkers in adult, immunocompetent, mixed viral-bacterial CAP show different inflammatory behavior than bacterial and viral CAP, as follows:

A) Mixed CAP had similar PCT levels to bacterial CAP but different from viral CAP, and CRP levels were higher in mixed than in either bacterial or viral CAP (Table 4). PCT and CRP levels were higher in mixed than overall viral and bacterial group, and than CAP of unknown cause (Table 4). These differences were not associated with a different severity.

B) PCT levels above 2.10 ng/mL on admission may indicate bacterial-involved pneumonia (bacterial or mixed viral-bacterial) with a high probability (78%). Elevated CRP levels was the only independent factor associated with mixed etiology, and a value below 25.95 mg/mL ruled out mixed etiology with 83% probability.

In recent years, some studies have shown the importance of mixed viral-bacterial pneumonia, reporting a prevalence of 4%-28% of CAP in adults, and 11%-39% of CAP with known etiology (1–7). These wide ranges are due to different study designs and, especially, to the number and type of diagnostic techniques used. In our study we included every test commonly used in clinical practice, as well as two different PCR assays for respiratory viruses, as these techniques are not standardized, in order to obtain the most complete virus yield possible. By following this protocol, we were able to identify a very definite group of mixed pneumonia and compare it to pure bacterial and viral groups.

The importance of mixed CAP has been recognized in recent years, and has been clearly underestimated so far. Some studies have found these types of CAP to be more severe than bacterial CAP [2,7,23], whereas others, like our study, found no differences in etiology-related severity [5,6]. The importance of suspecting mixed CAP is justified because these cases can be more severe and some of the viruses isolated can be treated.

Most mixed bacterial-viral CAP had pneumococcus as the causative agent, and half of our *S pneumoniae* pneumonias were associated with viruses. These data indicate that many cases of CAP usually considered pneumococcal CAP, are in reality mixed viral-bacterial infections, because viruses are not usually looked for. Conversely, when viral PCR is used, many considered as viral CAP can be in reality mixed, with missed bacteria [3].

It is true that a viral genome may be isolated from the upper airways of some asymptomatic subjects and, therefore, in patients with CAP, this may suggest previous or concomitant upper respiratory viral infection unrelated to the pneumonia. A study including BAL in severe CAP found that a viral genome was identified in upper respiratory samples without its alveolar isolation in only 13% of cases [24], and we only identified a virus in 6.7% in our controls, similar to other studies [4]. Not all authors are clear as to whether viruses other than influenza can, by themselves, cause pneumonia in adults or whether they must act in conjunction with other respiratory pathogens [2,3,6-8]. Rhinovirus was the most common virus identified in BAL of patients with severe CAP in a recent study [24], and our data showing a different inflammatory response of pure viral pneumonia compared with those of bacterial-viral CAP also suggest that viruses, other than influenza, can, by themselves, cause pneumonia in adult patients.

There is information in the literature confirming that viral pneumonia induces a different biomarker response compared to bacterial pneumonia. In fact, other studies have confirmed these differences also including atypicals [11-13]. The lack of PCT response to viral disease seems to be due to stimulation of macrophages to release interferon, which inhibits tumor necrosis factor (TNF) synthesis that, in turn, is necessary for tissues to synthesize PCT [15]. In our study, PCT levels were similar in mixed and bacterial CAP, and higher than those of viral CAP, and in terms of CRP, we found that mixed CAP showed significantly higher levels than bacterial and viral pneumonias. This biomarker was the only independent factor associated with mixed etiology. The high NPV for CRP, can help us to suspect an aetiology different than mixed bacterial/viral. However, it is true that its low PPV and AUC limit its usefulness in the clinical setting.

Some studies have suggested a potential role of biomarkers in differentiating pandemic influenza A H1N1 CAP from its bacterial coinfection. All these studies were retrospective and involved a limited number of mainly critical (ICU) patients (the largest series included 19 cases of mixed CAP), and found lower levels of PCT [16] and of PCT and CRP [18,19] in viral CAP than mixed H1N1-bacterial CAP, as well as their utility in clinical practice [16,18,19]. However, others showed limited sensitivity and specificity [17]. We also found a serum CRP higher in mixed infections involving influenza, but differences could not be achieved for PCT, probably due to low number of our cases (n = 15). With the exception of influenza, there is no information in adults on the behavior of biomarkers in mixed pneumonia. In our study, we found that the inflammatory response was higher in mixed CAP compared to single bacterial and viral CAP. We included mixed viral-bacterial CAP not restricted to the influenza virus in a homogeneous adult group of patients, and both PCT and CRP were different in viral CAP without influenza than mixed CAP without influenza.

The ability of CRP to discriminate mixed viral-bacterial CAP from bacterial CAP was considered limited in children [13], and the CAPNETZ group showed 58 cases of mixed CAP in adults that included two or more typical or atypical bacteria, and combinations of typical with atypical and any bacteria with virus, in which PCT and CRP levels were tested together [10]. Although increased amounts of biomarkers, especially PCT, have been associated with increased pneumonia severity [10], this was not the case in our study, as we found no differences in either PSI or CURB56 scores among three etiological groups. Median CRP was highest in mixed CAP, perhaps reflecting an increased level of systemic inflammation, as has been suggested [7]. It is difficult to explain with accuracy why mixed CAP showed higher CRP levels than both viral and bacterial pneumonias. It has recently been suggested that the main etiological agents of CAP present different inflammatory profiles, according to their respective biomarker (CRP, PCT, TNF-α, and IL-6) response, and that host-microorganism interplay may be useful for etiological diagnosis [11]. Our results suggest that mixed bacterial/viral CAP have a predominant CRP response. Findings of investigations approaching usefulness of PCT and CRP for etiological diagnosis of CAP are contradictory [25]. This can be due, at least in part, to the lack of inclusion of mixed bacterial/viral pneumonias in these studies.

There were no differences in WBC counts among the three etiological groups.

Our study is subject to certain limitations. This is a study from a single hospital, with a limited number of patients. We obtained no lower respiratory tract samples (BAL) for viral study, and some of the nasopharyngeal viral findings may have come from concomitant viral upper respiratory infection. We did not use PCR techniques for diagnosing atypical bacteria, and our yield for these pathogens, based in serological testing, was low. Nevertheless, our purpose was to study viruses and bacteria, and atypical findings were excluded. We are aware that some pneumonias classified as viral could, in fact, have been mixed, with a bacteria that could not be identified without a quantified PCR test for bacteria. Finally, a sequential search for biomarkers might have given us more complete information. The major strength of our study is that we compared a very well defined population of mixed CAP to other groups of very well defined bacterial and viral CAP.

## Conclusions

Our findings suggest a specific inflammatory profile in mixed viral-bacterial CAP, which is different from that of both bacterial and viral CAP. These findings can be useful by clinicians to include antiviral treatment especially during the influenza season, and to suspect a bacterial role in the case of either symptoms suggesting virus involvement or viral isolation. Interestingly our results were similar when we excluded mixed influenza cases. This is the first study to assess biomarkers in a group of mixed viral-bacterial CAP other than influenza, and compared with bacterial and viral adult CAP. The biomarker profile in this group is different from that of viral CAP. This suggests that the inflammatory response to viruses, even excluding influenza, is different from that in mixed CAP and, therefore, that viruses do not always require a bacterial co-pathogen to produce pneumonia in adults. This may help us better understand the true role of viruses in CAP and, perhaps, encourage the development of effective antiviral drugs [8]. Despite their usefulness in some particular cases, we appear to lack an accurate biomarker to separate bacterial-involved CAP from viral CAP in clinical practice.

## Abbreviations

CAP: Community-aquired pneumonia; PCT: Procalcitonine; CRP: C Reactive Protein; WBC: White blood cells; NIPIA: Near-infrared particle immunoassay; TRACE: Time-resolved amplified cryptate emission; PCR: Polymerase chain reaction; DFA: Direct fluorescence antibody assay; ADV: Adenovirus; hMPV: Human metapneumovirus; RSV: Respiratory syncytial virus.

## Competing interests

The authors declare that they have no competing interests

## Authors’ contributions

Conception and design: SB, EM, RM, AT. Acquisition of data: SB, EM, SF, ABL, MAR, ALS, CP, RM, AT. Analysis and interpretation of data: CL, RM, AT, ABL, SB. Drafting or revising the article: SB, EM, SF, ABL, CL, MAR, ALS, CP, RM, AT. Final approval of the manuscript: SB, EM, SF, ABL, MAR, ALS, CP, CL, RM, AT.

## Pre-publication history

The pre-publication history for this paper can be accessed here:

http://www.biomedcentral.com/1471-2466/14/123/prepub
